# From Antibiotic Resistance to Antibiotic Renaissance: A New Era in Helicobacter pylori Treatment

**DOI:** 10.7759/cureus.36041

**Published:** 2023-03-12

**Authors:** Prabhav Kashyap Godavarthy, Chandra Puli

**Affiliations:** 1 Medicine, Mamata Academy of Medical Sciences, Hyderabad, IND; 2 Gastroenterology and Hepatology, London Gastro Care, Hyderabad, IND

**Keywords:** h. pylori, drug susceptibility, antibiotic resistance, h. pylori treatment challenges, anti-h. pylori therapy, h. pylori gastritis, drug regimens for h. pylori, article review, drug susceptibility testing and antibiotic resistance, helicobacter pylori

## Abstract

*Helicobacter pylori* (*H. pylori*) is a common bacterial infection that can cause gastric diseases, including gastric cancer. The standard treatment for *H. pylori* infection is a combination of antibiotics and acid suppressants, but antibiotic resistance has become a significant problem that can reduce treatment efficacy. The development of novel treatment strategies for *H. pylori* is necessary to reduce the burden of this infection on public health. This review article examines *H. pylori* antibiotic resistance and future treatment possibilities. We discuss transitioning from trial and error to antimicrobial stewardship and using multi-strain probiotics as an adjunct therapy for *H. pylori* eradication.

This review article provides an extensive overview of *H. pylori* antibiotic resistance and future treatment possibilities. It begins with an introduction and background of the topic, followed by a literature review. The review of the literature covers the transition from trial and error to antimicrobial stewardship, the diagnosis and treatment of *H. pylori*, the comparative effectiveness of multiple first-line treatment regimens, the ideal *H. pylori* treatment for the present and future, the use of probiotics to reduce adverse events and improve eradication rates, and the use of novel and effective therapeutic regimens in an era of increasing antibiotic resistance. The conclusion summarizes the review's findings and provides recommendations for future research. The terms *Helicobacter pylori*, infection, antibiotic resistance, clarithromycin, macrolide resistance, proton pump inhibitors, vonoprazan, triple regimen, susceptibility, and stewardship were used in a PubMed literature search.

Finally, the article highlights the urgent need for a global collaborative approach toward tackling antibiotic resistance in *H. pylori* infections.

## Introduction and background

*Helicobacter pylori* (*H. pylori*) is a spiral-shaped gram-negative bacterium that can colonize the human stomach and cause chronic gastritis, peptic ulcer disease, mucosa-associated lymphoid tissue lymphoma, and gastric cancer [1]. *H. pylori* infection is one of the most common chronic infections worldwide, with an estimated prevalence of 50% in developing countries and 10-20% in developed countries [2]. The World Health Organization (WHO) has identified *H. pylori* as a global priority pathogen due to its increasing prevalence and the emergence of antibiotic resistance [3].

The WHO has identified *H. pylori* as one of the 12 most critical antibiotic-resistant bacteria that should be prioritized for developing new antibiotics [3]. The increasing prevalence of antibiotic resistance to *H. pylori* has made the treatment of this infection more complex and led to the need for new and effective treatment strategies. In this review article, we will discuss the current state of *H. pylori* antibiotic resistance and future treatment possibilities.

The history of *H. pylori* treatment has evolved over the past few decades. In the 1980s, *H. pylori* was discovered as the causative agent of peptic ulcer disease, and treatment initially consisted of acid suppression therapy alone [1]. Later, it was discovered that antibiotics could be used to eradicate *H. pylori*, and a combination of antibiotics and acid suppression therapy became the standard treatment for *H. pylori* infection. However, antibiotic resistance has become a significant problem, and the efficacy of treatment has declined in recent years. The bacterium can be eradicated with a combination of antibiotics and acid suppressants, but antibiotic resistance has become a significant problem that can reduce the efficacy of treatment [4].

The diagnosis and treatment of *H. pylori* infection have been the focus of several consensus statements and guidelines. Treatment of *H. pylori* infection typically involves the use of antibiotics, and the most common regimens are triple therapy and quadruple therapy [5]. However, the increasing prevalence of antibiotic resistance has led to an increased failure rate of *H. pylori* eradication. To combat the emergence of antibiotic resistance in *H. pylori*, there has been a paradigm shift in *H. pylori* therapy, transitioning from trial and error to antimicrobial stewardship. Antimicrobial stewardship is an approach that aims to optimize the use of antimicrobial agents to reduce the development of antimicrobial resistance and preserve the efficacy of antimicrobial agents [6].

Methods

Search Strategy

A comprehensive literature search was performed using the PubMed database for studies published until 2022. The following keywords were used: *Helicobacter pylori*, infection, antibiotic resistance, clarithromycin, macrolide resistance, proton pump inhibitors, vonoprazan, triple regimen, susceptibility, and stewardship. Only studies in the English language were included.

Data Screening

The inclusion criteria were studies that evaluated *H. pylori* treatment guidelines, antibiotic resistance, and diagnosis, and studies that investigated the use of probiotics as an adjunct therapy for *H. pylori* eradication. The exclusion criteria were studies that focused on other bacteria, studies that evaluated the efficacy of alternative non-antibiotic therapies, studies published in non-peer-reviewed journals, and studies that were not relevant to the topic. Initially, 89 studies were identified based on the search criteria. After screening titles and abstracts, 50 studies were excluded because they were not relevant to the topic. The remaining 39 studies were reviewed in full-text form. Out of these, 14 studies were excluded because they did not meet the inclusion criteria. Finally, a total of 24 studies and one website reference were included in this narrative review.

Data Extraction and Synthesis

Data were extracted from each study that included information on study design, sample size, intervention, outcome measures, and conclusions. The extracted data were synthesized narratively, and the findings were summarized and discussed.

## Review

Helicobacter pylori infection

*H. pylori* is a gram-negative, spiral-shaped bacterium that colonizes the stomach and is associated with various gastrointestinal diseases, including gastritis, peptic ulcer disease, and gastric cancer [1]. *H. pylori* infection is one of the most common chronic infections worldwide, with an estimated prevalence of 50% in developing countries and 10-20% in developed countries [2]. The prevalence of *H. pylori* infection varies by region, with higher prevalence in developing countries and lower prevalence in developed countries [7].

Diagnosis of H. pylori and current treatment guidelines

Diagnosing *H. pylori* infection is usually based on serological tests, breath tests, stool antigen tests, and endoscopy with biopsy [5]. The treatment of *H. pylori* infection consists of a combination of antibiotics, proton pump inhibitors (PPIs), and sometimes bismuth compounds. The most commonly used antibiotics for treating *H. pylori* infection are clarithromycin, amoxicillin, metronidazole, and tetracycline [8].

The diagnosis of *H. pylori* infection is typically based on a combination of clinical, endoscopic, and laboratory tests, such as the urea breath test, stool antigen test, and serology [9-11]. Recent advances in *H. pylori* diagnostics include the use of next-generation sequencing [10].

The diagnosis and treatment of *H. pylori* infection have been the focus of several consensus statements and guidelines, including the Kyoto global consensus report on *H. pylori* gastritis [12], the Houston consensus conference on testing for *H. pylori* infection in the United States [13], and the Taipei global consensus [14].

Treatment of *H. pylori* infection typically involves the use of antibiotics, and the most common regimens are triple therapy and quadruple therapy [5]. Triple therapy typically involves a PPI and two antibiotics (clarithromycin and amoxicillin or metronidazole). Quadruple therapy typically consists of PPI, bismuth, metronidazole, and tetracycline.

Current worldwide recommendations and a network meta-analysis [8] evaluating the effectiveness of various treatment regimens advocate vonoprazan-based triple therapy for seven days and non-bismuth quadruple therapies for 10-14 days. Vonoprazan triple therapy and reverse hybrid therapy - a hybrid (dual-quadruple) therapy consisting of a PPI and amoxicillin for 14 days with the addition of clarithromycin and metronidazole for the final seven days - achieved high eradication rates of >90% for first-line *H. pylori* infection. Levofloxacin triple therapy produced the highest eradication rates in Western nations. The standard triple treatment regimen was the least effective in the network meta-analysis. Due to the empiric use of numerous antibiotics, these regimens still have intrinsic flaws that could encourage additional rises in antimicrobial resistance and create gut microbiota dysbiosis.

Effectiveness of multiple different first-line treatment regimens for Helicobacter pylori infection

A network meta-analysis was conducted to compare the effectiveness of multiple first-line treatment regimens for *H. pylori* infection [8]. The analysis included 18 randomized controlled trials with a total of 6,744 patients. The results of the analysis showed that the most effective treatment regimens were those containing a PPI, amoxicillin, clarithromycin, and metronidazole. The analysis also showed that adding bismuth compounds to the treatment regimen had no significant effect on the eradication rate.

The choice of the first-line treatment regimen for *H. pylori* infection depends on various factors, including the local prevalence of antibiotic resistance and patient factors such as age, comorbidities, and medication allergies.

The standard first-line therapy for *H. pylori* eradication consists of a triple therapy regimen, which includes a PPI, clarithromycin, and either amoxicillin or metronidazole [8]. However, the success rates of this regimen have declined due to the emergence of antibiotic-resistant strains of *H. pylori*. According to a network meta-analysis of multiple first-line treatment regimens for *H. pylori* infection, the most effective regimen was bismuth quadruple therapy, which includes a PPI, bismuth, tetracycline, and metronidazole [8]. However, this regimen is associated with more side effects and is more complicated to administer than triple therapy. In regions with high clarithromycin resistance rates, levofloxacin-containing triple therapy or bismuth quadruple therapy are recommended as first-line therapy [8]. In addition, sequential therapy, which includes a PPI and amoxicillin for the first five days, followed by a PPI, clarithromycin, and metronidazole for the next five days, has been proposed as an alternative to standard triple therapy [8].

Antibiotic resistance in H. pylori

Most of the treatments for *H. pylori* on the market today have cure rates of about 80% or less. Ineffective treatment regimens, uncooperative patients, high gastric acidity, internalizing bacteria, gene polymorphisms (interleukin-1B and CYP2C19), antimicrobial washout and dilution, biofilm development, and, most importantly, antibiotic resistance are all factors contributing to ineffective eradication of *H. pylori* [1]. Most treatment recommendations are based on comparisons of randomized trials that emphasize relative rather than absolute effectiveness, and the actual trials included in these meta-analyses typically differ markedly in the specifics of dosing, the relative potency of the PPI used, and the prevalence of resistance in the populations. Due to these restrictions, the suggested treatments frequently fall short of achieving acceptable cure rates. Treatment guidelines for other infectious diseases are frequently susceptibility-based and created to consistently attain predetermined cure rates, such as consistently greater than 90% or 95% [15].

The development of antibiotic resistance in *H. pylori* is a significant challenge in the treatment of this infection. The WHO has released a list of 12 bacterial families that are the most dangerous to human health as "priority pathogens," categorizing them into three priority statuses: critical, high, and medium [3]. Clarithromycin-resistant *H. pylori* is in the same “high priority” category as methicillin-resistant *Staphylococcus aureus* and vancomycin-resistant *Enterococcus faecium*. Moreover, *H. pylori* strains resistant to fluoroquinolones and metronidazole, which are frequently employed in eradication therapies, have risen to worrying levels (in excess of 15%) in many regions of the world [16]. The primary antibiotics used to treat *H. pylori* infection are clarithromycin, metronidazole, and amoxicillin, but resistance to these antibiotics has become increasingly common [5]. Antibiotic resistance has made it so that triple treatments with clarithromycin, metronidazole, or fluoroquinolone can no longer be used on a whim.

Clarithromycin and/or metronidazole are also included in the currently advised regimens of vonoprazan-based triple therapy and non-bismuth quadruple therapy, both of which have significant issues with rising antimicrobial resistance.

The increasing prevalence of antibiotic resistance has led to an increased failure rate of *H. pylori* eradication [6]. Studies have shown that *H. pylori*'s resistance to antibiotics is increasing globally.

Clarithromycin resistance is a particular concern, as it is a key component of first-line therapy for *H. pylori*. The prevalence of clarithromycin resistance varies widely by geographic region, with higher rates reported in some Asian countries and lower rates in Europe and North America [6]. In a recent study in Japan, the eradication rate for clarithromycin-resistant *H. pylori* was only 47.4%, compared to 89.6% for clarithromycin-susceptible strains [17]. Metronidazole resistance rates are also high, ranging from 40% to 70% worldwide [8]. Tetracycline resistance is relatively low, but its side effects limit its use [8]. Levofloxacin is effective against *H. pylori*, but its use is limited by the development of resistance [8]. Amoxicillin resistance is low, and still, it is an effective antibiotic for *H. pylori* treatment [8].

It has been found that the eradication rates of *H. pylori* have declined over time, and the efficacy of treatment with standard triple therapy has decreased to below 80% in many regions [8]. The declining efficacy of standard treatment regimens highlights the urgent need for novel treatment strategies for *H. pylori* infection.

In Europe, the prevalence of antibiotic resistance was found to be higher in countries with higher antibiotic consumption [18].

Previous exposure to macrolide antibiotics has been found to be associated with an increased risk of treatment failure in patients with *H. pylori* infection [19,20].

The transition from trial and error to antimicrobial stewardship

The traditional approach to treating *H. pylori* has been a trial-and-error approach, which involves the use of multiple antibiotics in combination with acid-suppressing medications. This approach is associated with a high rate of failure due to the increasing prevalence of antibiotic resistance [1]. To address this issue, a new approach to *H. pylori* therapy has been proposed, which is based on antimicrobial stewardship. Antimicrobial stewardship is a systematic approach to the use of antibiotics that involves the selection of the most appropriate antibiotic for a given infection, optimizing the dosage and duration of treatment, and monitoring patient outcomes [15]. The goal of antimicrobial stewardship is to optimize the use of antibiotics to reduce the emergence of antibiotic resistance and improve clinical outcomes [6]. Excluding antibiotics where pre-existing resistance is expected is the first step in finding and prescribing an effective therapy. History and/or susceptibility testing can be used to achieve this. A complete history of previous antibiotic use should be taken, together with a review of any relevant medical and, if available, pharmacy data [5].

The use of probiotics and Lactobacillus to reduce adverse events and improve eradication rates

Probiotics are live microorganisms that can provide health benefits when consumed adequately [21]. Probiotics (mainly *Lactobacillus*, *Saccharomyces boulardii*, and *Bacillus clausii*) have been shown to reduce the side effects of *H. pylori* eradication therapy and improve the eradication rates [21-23]. It has been demonstrated [2] that probiotics can effectively enhance the eradication rate of *H. pylori* and reduce the incidence of antibiotic-associated adverse effects such as diarrhea and abdominal pain [2,21,23]. In a meta-analysis of randomized controlled trials [2], it was found that the use of four probiotic combinations (*L. acidophilus*/*B. animalis*, *L. helveticus*/*L. rhamnosus*, *L. acidophilus*/*B. longum*/*E. faecalis*, and the eight-strain mixture) was significantly effective (eradication rates higher than 90%) as an adjunct therapy for the eradication of *H. pylori* (OR: 1.53, 95% CI: 1.23-1.90) and a substantial reduction in the incidence of antibiotic-associated side effects (OR: 0.54, 95% CI: 0.40-0.72). However, the optimal strains and dosages of probiotics have yet to be established, and further research is needed to determine the most effective probiotic regimens. Multi-strain probiotics were utilized in another trial [24] over a prolonged period (three to five weeks) and at high doses (more than 1010 CFU/day), and the multi-strain probiotics had >90% eradication rates. In a randomized, double-blind, placebo-controlled trial, *B. clausii* therapy was found to reduce the side effects of anti-*H. pylori* treatment, such as diarrhea and abdominal pain [22].

The use of novel and effective therapeutic regimens in an era of increasing antibiotic resistance

In an era of increasing antibiotic resistance, novel and effective therapeutic regimens are needed to treat *H. pylori* infection. These regimens should consider factors such as the patient’s age, comorbidities, and antibiotic resistance [24]. In addition, they should be based on antimicrobial stewardship principles to reduce the emergence of antibiotic resistance [25]. The ideal treatment for *H. pylori* infection should be effective, safe, and cost-effective. It should also be tailored to the individual patient, considering factors such as age, comorbidities, and antibiotic resistance [4]. In addition, the treatment should be based on antimicrobial stewardship principles to reduce the emergence of antibiotic resistance.

Quadruple therapy and *Lactobacillus*-containing quadruple therapy have been proposed as novel and effective therapeutic regimens for *H. pylori* infection [16,24]. Quadruple therapy consists of PPI, bismuth, metronidazole, and tetracycline and is more effective than triple therapy for *H. pylori* eradication [8]. *Lactobacillus*-containing quadruple therapy consists of a PPI, bismuth, metronidazole, and a probiotic containing *Lactobacillus* and has been shown to be more effective than quadruple therapy for *H. pylori* eradication [8].

Another potential solution is using non-antibiotic therapies, such as bismuth-containing regimens and vaccines. Bismuth-containing regimens are effective in the treatment of *H. pylori* and may have a role in the treatment of antibiotic-resistant strains [1]. In addition, several *H. pylori* vaccines are being developed and have shown promising results in preclinical studies [24]. However, further research is needed to determine the safety and efficacy of these non-antibiotic therapies in clinical trials.

## Conclusions

In conclusion, antibiotic resistance contributes to lower eradication rates and higher rates of treatment failure when dealing with *H. pylori* infection. The emergence of antibiotic-resistant strains has brought into stark relief the need for new therapeutic approaches. Antimicrobial stewardship principles should form the basis of this strategy to slow the spread of antibiotic resistance. Furthermore, since they do not require unnecessary antibiotics, susceptibility-based therapies provide options in a time of rising antibiotic resistance. Future research should focus on developing novel and effective therapeutic regimens for *H. pylori* infection in an era of increasing antibiotic resistance. The effectiveness and safety of innovative treatment plans for *H. pylori* infections, such as quintuple therapies, high-dose dual treatments, and conventional triple therapy with probiotics, are still being studied in some trials. Although some encouraging findings have been reported, there is not enough support for the claims.

Potential options include susceptibility-guided therapy, non-antibiotic therapies, and probiotics; however, further study is needed to determine their safety and efficacy in clinical trials. As the prevalence of antibiotic-resistant *H. pylori* rises, creating new medications and exploring other treatment methods may become necessary. Antibiotics should not be used in regimens if they are not necessary because doing so could affect their effectiveness and exacerbate the resistance situation. To effectively manage *H. pylori* infection and prevent antibiotic resistance, a holistic approach will be required that makes use of both existing and developing therapeutic options in addition to adopting antimicrobial stewardship initiatives.
